# Twelfth Annual Meeting of the Indiana State Dental Association

**Published:** 1870-08

**Authors:** Seneca B. Brown


### Proceedings of Societies.

---

### TWELFTH ANNUAL MEETING OF THE INDIANA STATE DENTAL ASSOCIATION.

The twelfth annual meeting of the Indiana State Dental Association, convened at 2 o'clock, P. M., June 28, 1870, in the lecture room of the Indiana Medical College, at Indianapolis.

Dr. Jno. F. Johnston, President, called the Association to order, and directed the reading of the minutes of the eleventh annual meeting, held last June, which were adopted.

The roll was then called, and the following members responded:

A. M. Moore, Lafayette; A. T. Keightley, Greencastle; W. E. Driscoll, Bedford; E. V. Burt, Lafayette; W. F. Morrill, New Albany; E. M. Morrison, Noblesville; I. Knapp, Fort Wayne; J. W. Ellis, Newcastle; A. O. Rawls, Connersville; Seneca B. Brown, Fort Wayne; Jno. F. Johnston, P. G. C. Hunt, W. S. Heiskill, Merritt Wells, Indianapolis.

The President appointed the following Board of Censors: Drs. I. Knapp, A. M. Moore, W. F. Morrill.

Subsequently the Board reported favorably on the applications of the following:

S. T. Kirk, Kokomo; C. C. Burns, Greensburg; J. W. Hollinsworth, Mt. Vernon; J. S. Rice, Shelbyville; L. H. Pumphrey, Princeton.

These gentlemen were unanimously elected members of the Association, by separate ballot. The Treasurer of the Association (C. C. Burgess,) being absent, by consent, E. M. Morrison was appointed Treasurer, *pro tem.*

The newly elected members were now formally introduced to the Association, by President Johnston.

By consent, the order of business was dispensed with, and Dr. Hollingsworth was invited to address the Association, on the process of Casting Aluminum Base, as discovered by himself.

He accordingly explained his method and exhibited specimens of work.

On motion, the Association adjourned till 8 o'clock P. M.

EVENING SESSION.

President Johnston resumed the chair at 8 o'clock, and called the Association to order. Minutes of afternoon session read and approved.

The first subject for discussion being "Hypodermic Medication as it pertains to Dentistry," being now in order, President Johnston called upon W. F. Morrill for an essay upon this subject, which he had been appointed to prepare for the opening of the discussion.

Dr. Morrill then read a carefully written paper on the subject assigned him.

On motion, Prof. Judd of St. Louis, was elected an honorary member of the Association.

Prof. Judd being called upon to take part in the discussion, related several interesting cases that had come under his observation.

The President here remarked that much valuable information presented in our discussions, were lost for want of a verbatim reporter, and as a short-hand reporter was present, he thought proper for members to consider the matter.

On motion, by Dr. Knapp, it was

*Ordered*, That W. H. Drapier be hereby appointed official reporter of this Association.

The discussion of the subject under consideration, was then continued by Drs. Hunt, Morrill, Moore, Knapp, Judd and Rawls.

The President appointed Prof. Moore, to act as Clinical demonstrator, at 8 o'clock, A. M., to-morrow, in the rooms of Strong & Smith.

Dr. Morrill moved an adjournment till to-morrow morning, at 9 o'clock, which was agreed to.

### SECOND DAY.

#### *Morning Session.*

Association met at 10 o'clock. President Johnston in the chair. Minutes read and approved.

On motion, the pending order of business was dispensed with, and the 6th subject, "Miscellaneous," was taken up in order to accommodate a member who desired to leave, and wished to take part in this subject. Accordingly, Dr. Keighley, of Greencastle, opened the discussion, on "The best substitute for Vulcanized rubber as a base for Artificial Teeth." The Doctor has a process of his own for the preparation of Aluminum, which metal he prefers.

Dr. Phemster, of Greencastle, also described his process for casting Aluminum, and regards it as the best substitute for rubber.

Prof. Watt, of Cincinnati, regarded Aluminum as much better than rubber; was of the opinion, however, that it would not prove to be entirely satisfactory. His experiments had been made with a view to get something more easily manipulated than Aluminum, so simple as to induce even tyros to abandon the use of rubber, and thought he had succeeded in obtaining a metal that is about as easily cast into Dental plates. It is known as Watt & Williams' Improved Alloy. This alloy successfully resists sulphur, oxidation and chloridation; was of the opinion that humanity had been poisoned long enough with that infernal rubber nuisance.

Dr. Phemster thinks in all the 69 elements, nothing is so admirably adapted, on account of lightness, strength and purity, as Aluminum. Dr. Stanley spoke in faver of Watt

& Williams' alloy, because it retains its color better, if any odds, than Aluminum.

Prof. Judd of St. Louis, had been as anxious to see rubber go out of use as any one, for several reasons, the first and greatest being because satisfied, after thorough investigation, that thousands of people are annually poisoned with it. Thought Aluminum withstands the juices of the mouth perfectly, and is vastly superior to rubber in all shapes and forms.

Dr. W. F. Morrill observed a deposit of lime and other secretions from the mouth on Aluminum plates, when they had been worn any length of time, even where particular care has been exercised. As a substitute for rubber, he recommended Rose Pearl. It can be made more thin, it is lighter than Aluminum. In color, a close imitation to nature—it is nearly as cheap as rubber, and thinks it is bound to come into general use sooner or later.

On motion, the discussion was closed on the subject.

The Second subject: "What are the Indications for Destroying the Pulp," was now declared in order. Dr. Knapp, who had by appointment prepared an essay to open the discussion, came forward and read the same.

On motion, the thanks of the Association were tendered him for his very meritorious paper, and a request made for its publication.

L. S. Carter, of Dunleith, was proposed and duly elected a member.

Adjourned to meet at 2 o'clock P. M.

### AFTERNOON SESSION.

At 2 o'clock, President Johnston resumed the chair. Minutes read and approved. The Association proceeded with the discussion of the question pending at the time of adjournment for dinner.

Prof. Taft, of Cincinnati, contended earnestly for the saving of the pulps of the teeth.

Prof. Judd, of St. Louis, Prof. Watt, of Cincinnati, Prof. Moore, of Lafayette, President Johnston, Drs. Knapp, Burgess, Morrill and Rawles, all followed in the same direction.

On motion, the discussions were then closed, and

The Third subject for discussion, viz: "Diseases of the Gums, Alveolus, and Treatment," taken up.

The President announced that Dr. S. M. Good had been appointed to open the discussion, but a letter was received from him, stating that it was impossible for him to be present.

On motion of Dr. Knapp, the subject was passed over informally.

The President announced the Fourth subject, to-wit: "Filling Teeth with Gold." Prof. Moore, of Lafayette, to open the discussion.

Prof. Moore read his essay in which he described minutely the process of filling teeth, illustrating his ideas with a very fine specimen of his workmanship.

Dr. Morrill moved that the thanks of the Association be tendered Prof. Moore for his paper, and that it be published with the transactions of this meeting.

Motion was agreed to.

On motion of Dr. Knapp, it was

*Ordered*, That the discussion of Prof. Moore's paper be the order till five and a half o'clock, at which time the Association will go into the election of officers for the ensuing year, when the Association is to adjourn to the dissecting room to witness Dr. Hollingsworth's method of casting Aluminum.

Profs. Judd and Taft, and Dr. Hunt proceeded with the discussion.

The election of officers for the ensuing year, being next in order, Prof. Moore nominated W. C. Stanley, of Dublin, for President.

Drs. Moore, Johnston, Brown and Hunt were also nominated, but they severally declined the honor.

On motion of Dr. Hunt, the Secretary was authorized to cast the vote of the Association for Dr. Stanley, this having been done in the case of all the officers elected.

The President severally announced the result as follows:

President—W. C. Stanley, of Dublin.

1st. Vice President—E. V. Burt, of Lafayette.

2d. Vice President—W. S. Heiskill, of Indianapolis.

Treasurer—C. C. Burgess, Indianapolis.

Secretary—Seneca B. Brown, Fort Wayne.

The retiring President then delivered his address, and as his last official act, appointed Drs. Hunt and Moore to escort the President elect to the chair.

Prof. Moore introduced President Stanley, who thanked the Association for the honor conferred.

On motion of Dr. Johnston, the association agreed to reassemble at 8 o'clock P. M. The members then repaired to the anatomical lecture room, and witnessed Dr. Hollingsworth's cast Aluminum plate, by his process.

### EVENING SESSION.

President Stanley took the chair at 8½ o'clock. Reading of the minutes was dispensed with.

The President announced the following committees:

*Auditing Committee*—Dr. W. F. Morrill, Prof. A. M. Moore.

*Executive Committee*—Drs. M. Wells, E. M. Morrison, I. W. Ellis and I. Knapp.

On motion of Dr. Johnston, the regular order of business was suspended, and "Miscellaneous Business" taken up. He then submitted the following resolution which was unanimously adopted:

*Resolved*, That the thanks of this Association are hereby tendered to the trustees and faculty of the Indianapolis Medical College, for their courtesy and kindness in permitting us the free use of their lecture room.

Dr. Johnston read the resignation of Lea W. Munhall as a member of this Association, dated February 2, 1870, and moved that it be accepted.

Dr. Hunt was under the impression that such a motion was not contemplated in the Constitution.

As there was no power to accept his resignation, since its date till now, and as Dr. Munhall had while a member of this Association, violated Art. 2, Sec. 3, of its Code of Ethics, and as it was not his first offense, expulsion was the only course the Association could pursue.

Dr. Morrill, chairman of the committee to inquire into infractions of the Dental Code of Ethics, presented a report on Dr. Munhall's case. Dr. Johnston withdrew his motion recommending expulsion. The report was then concurred in, and the President declared that Dr. Munhall was expelled.

The auditing committee reported that they had examined and found correct the Treasurers report, viz :

| | |
|---|---|
| Amount cash on hand | $71 64 |
| Amount received initiation fees | 30 00 |
| Amount annual | 32 00 |
| Amount on hand | $133 64 |

C. C. Burgess, Treasurer.

Adopted.

A. M. Moore,

W. F. Morrill. } Aud. Com.

C. C. Burgess moved that when the Association adjourn, it be till the last Tuesday in June, 1871, to meet in Fort Wayne. Agreed to.

On motion of Dr. Johnston, it was

*Ordered*, That the President and Secretary be authorized to issue certificates as delegates to the National Dental Association, to be held at Nashville, to all members of the Association who may apply, not exceeding the limited number.

On motion, Prof. Moore, J. F. Johnston and P. G. C. Hunt, were authorized to negotiate for reduced fare for delegates attending the National Association.

On motion of Dr. Knapp, Drs. Johnston and Hunt were appointed to select from the report taken by the official reporter, such portions of the proceedings as in their judgment would be valuable for publication in the *Dental Journals and Indiana Journal of Medicine.*

On motion by Dr. Morrill, the Association proceeded to the consideration of the Fifth subject, to-wit: "Treatment and Preservation of the Deciduous Teeth."

Dr. Morrison having been appointed to open this discussion, read an essay, which received the thanks of the Association, and a copy was requested for publication.

On motion of Dr. Knapp, the discussion of his paper was suspended for

Dr. W. E. Driscoll, who read a paper on the "Elevation of the Dental Profession," for which the thanks of the Association were tendered, and a copy requested for publication.

Dr. Johnston read a letter from Dr. M. S. Dean, of Chicago, refering to the testimonial fund for Dr. S. C. Barnum, in acknowledgment of his gift to the profession, viz: the rubber dam.

On motion, the Association proceeded to the consideration of Dr. Morrison's paper. Remarks were made by Prof. Judd, Prof. Taft, Prof. Watt, and Dr. Knapp.

The Association then adjourned.

SENECA B. BROWN, Sec.

